# Epitope spreading in multiple sclerosis is shaped by the spatial organisation of immune responses

**DOI:** 10.3389/fimmu.2026.1878029

**Published:** 2026-07-28

**Authors:** Aleksandra Rutkowska

**Affiliations:** Department of Anatomy and Neurobiology, Rutkowska Lab, Medical University of Gdańsk, Gdansk, Poland

**Keywords:** 7α,25-Dihydroxycholesterol (7α,25-OHC), B cells, EBI2 (GPR183), epitope spreading, Epstein–Barr virus, germinal centres, immune cell migration, multiple sclerosis

## Abstract

Epitope spreading has long been proposed as a mechanism contributing to the progression of multiple sclerosis (MS), yet its role in human disease remains incompletely defined. Traditionally viewed as a consequence of antigen release during tissue damage, epitope spreading is often considered independently of the anatomical context in which immune responses occur. Here, I re-examine epitope spreading in MS by integrating evidence from experimental models and human studies with recent advances in neuroimmunology. I propose that the diversification of autoreactive responses is not solely driven by antigen availability but is shaped by the spatial organisation of immune cells within the central nervous system (CNS), CNS-draining lymph nodes, and peripheral lymphoid tissues. In particular, I highlight EBI2 (GPR183) as a candidate regulator of immune-cell positioning that may influence the cellular interactions required for the emergence of new antigen specificities. This spatial framework links epitope spreading with germinal-centre dynamics, B-cell-mediated immunity, and Epstein–Barr virus-associated mechanisms in MS, while emphasising that direct evidence connecting EBI2 to epitope spreading remains limited. Defining how autoreactive diversification is controlled may help explain disease progression and guide strategies aimed at limiting the evolution of pathogenic immune responses.

## Highlights

- Epitope spreading in multiple sclerosis is shaped by spatial organisation of immune responses.
- Antigen availability alone does not explain diversification of autoreactivity in MS.
- EBI2-dependent immune-cell positioning may influence interactions associated with autoreactive diversification.
- Germinal centre (GC) dynamics and B-cell responses contribute to the evolution of autoreactive repertoires.
- Therapeutic modulation of EBI2 (GPR183) presents a potential trade-off between limiting inflammation and promoting repair.

## 1 Introduction

Epitope spreading has been widely proposed as a mechanism underlying the progression of autoimmune diseases, including multiple sclerosis (MS). It describes the diversification of immune responses from an initial antigenic target to additional self-epitopes (1). Although this concept is well supported by experimental models, its role in human MS remains difficult to define, and it is often considered primarily as a consequence of antigen release during tissue damage. However, the anatomical context in which immune responses develop is rarely considered. The activation of autoreactive lymphocytes, their interaction with antigen-presenting cells, and the selection of new antigen specificities all depend on where and when these encounters occur. Epitope spreading has traditionally been discussed in the context of antigen release, molecular mimicry, and the progressive diversification of autoreactive immune responses. Likewise, the roles of EBV infection, B cells, germinal centre reactions, and EBI2-dependent immune-cell migration have largely been examined as separate components of MS pathogenesis. However, less attention has been paid to how the anatomical organisation of immune responses influences whether newly available antigens are incorporated into the autoreactive repertoire. Because antigen presentation, lymphocyte activation, and affinity maturation occur within defined tissue niches, the location of these interactions may be as important as antigen availability in shaping autoreactive diversification. In this Review, I therefore examine epitope spreading through a spatial framework that integrates evidence from the central nervous system (CNS), CNS-draining lymph nodes, and peripheral lymphoid tissues.

## 2 Epitope spreading as a mechanism of chronic CNS autoimmunity

The epitope spreading theory proposes that chronic tissue inflammation and damage promote autoimmunity by expanding immune responses from an initial dominant epitope to secondary self-epitopes (1). During tissue injury, previously cryptic determinants are released and taken up by antigen-presenting cells (APCs) within the inflamed environment. These epitopes are processed and presented to naïve T lymphocytes, resulting in the activation of new autoreactive T-cell populations that sustain tissue damage independently of the initiating antigen (2, 3).

Experimental autoimmune encephalomyelitis (EAE) models provide strong support for this mechanism. In proteolipid protein (PLP)139–151-immunised mice, T-cell responses are initially restricted to the priming epitope but subsequently extend to PLP178–191 during relapses (4). Induction of tolerance to the secondary epitope alone significantly reduces relapse rates despite persistent reactivity to the primary antigen, indicating that newly emerging specificities contribute directly to disease activity (4). These findings show that diversification of antigen recognition can sustain chronic autoimmune inflammation (5, 6).

Evidence for epitope spreading in human MS remains limited and heterogeneous. Although several studies have reported diversification of T-cell and antibody responses over time, these findings are not consistently observed across patient cohorts, and stable patterns of antigenic evolution have not been identified. As a result, the extent to which epitope spreading contributes to disease progression in individual patients remains uncertain. Several studies nevertheless support the possibility of ongoing antigenic diversification. Longitudinal analysis of T-cell responses in patients has shown a shift in antigen recognition over time, with decreased reactivity to initial targets and emergence of responses to additional myelin epitopes as disease progresses (7, 8). However, this pattern is not uniform across patients, and stable epitope hierarchies have not been identified (9). This variability complicates the interpretation of epitope spreading in human disease and presents a challenge for the development of antigen-specific therapies.

Early evidence of epitope spreading has also been reported in paediatric MS, where expansion of antibody reactivity was observed within months of disease onset and was associated with progression from monophasic demyelinating events to MS (10). These findings suggest that diversification of autoreactivity may occur early in the disease course rather than being restricted to later stages.

Relapses in experimental models and in MS have been associated with the expansion of Th1- and Th17-mediated responses to newly emerging epitopes (11). At the same time, epitope spreading is not exclusively pathogenic. Experimental studies indicate that spreading of immune responses can also contribute to the resolution of inflammation. In non-obese diabetic mice, induction of a Th2 response against a single β-cell antigen resulted in the expansion of regulatory responses to additional antigens, mirroring the pattern observed in pro-inflammatory epitope spreading (12). Similarly, IL-10-based interventions and blockade of IL-23 signalling suppress disease progression and limit the emergence of new autoreactive specificities (13).

These observations suggest that epitope spreading reflects a broader property of adaptive immunity rather than a strictly pathogenic process. In MS, the balance between inflammatory and regulatory spreading may determine whether disease progresses or resolves. Importantly, the dynamic and evolving nature of antigen recognition presents a major obstacle for therapies targeting single epitopes, as the relevant antigenic targets may change over time (14). This is consistent with the current view of MS as a continuous disease process in which immunological activity persists beyond clinically defined relapses, contributing to ongoing tissue damage.

## 3 Where epitope spreading occurs in MS

Epitope spreading requires not only antigen release but also efficient antigen presentation and recruitment of naïve lymphocytes into these sites. In MS and its experimental models, these processes occur across multiple anatomical compartments, including the CNS, CNS-draining lymph nodes, and secondary lymphoid tissues.

Evidence from experimental models indicates that the CNS itself is an active site of epitope spreading. In both relapsing EAE and Theiler’s murine encephalomyelitis virus-induced demyelinating disease (TMEV-IDD), activation of naïve myelin-specific T cells can occur directly within the CNS (15). This challenges the classical view that priming is restricted to peripheral lymphoid organs and suggests that local antigen presentation within inflamed tissue is sufficient to drive diversification of autoreactive responses.

Microglia and infiltrating macrophages play a central role in this process. In the inflammatory environment of TMEV-IDD, microglia acquire potent antigen-presenting capacity and contribute directly to myelin damage (16). In addition, dendritic cells (DCs) present within the CNS have been shown to efficiently activate autoreactive T cells, particularly a subset of CD11c^+^CD45^hi cells capable of driving PLP-specific responses (15). These findings indicate that the CNS is not only a target of immune attack but also a site where new autoreactive specificities can be generated.

CNS-draining lymph nodes represent another key site for epitope spreading. Surgical removal of cervical and lumbar lymph nodes reduces relapse severity in multiple EAE models and alters antigen-specific responses, demonstrating that these structures contribute to the expansion of autoreactive immunity (17). Importantly, intermolecular spreading has been shown to occur within these lymph nodes, with distinct antigen-specific responses emerging in different draining sites. This suggests that spatial separation of immune responses may influence the pattern and extent of epitope diversification.

Secondary lymphoid organs also contribute to epitope spreading by supporting interactions between APCs and naïve lymphocytes. In EAE, widespread inter- and intramolecular spreading involving both T cells and autoreactive B cells has been demonstrated, highlighting the contribution of humoral responses to antigen diversification (18). Consistent with this, increased anti-myelin antibody responses have been observed in relapsing EAE models, indicating that B-cell-mediated mechanisms may amplify spreading in certain disease contexts.

Importantly, epitope spreading is not universally required for chronic disease progression. In transgenic models expressing a restricted myelin-specific T-cell receptor repertoire, relapses and chronic disease can occur in the absence of detectable spreading (19). These findings indicate that epitope spreading should be viewed as one mechanism capable of amplifying and diversifying autoreactive responses rather than as an obligatory driver of disease progression. Additional pathogenic processes can clearly sustain CNS autoimmunity even when diversification of antigen specificity is limited.

Finally, antigen presentation during EAE is not limited to professional APCs. Oligodendrocytes have been shown to present myelin antigens and contribute to the activation of CD8^+^ T cells, indicating that resident CNS cells may participate directly in determinant spreading (20). Together, these observations support a model in which epitope spreading emerges from coordinated interactions between resident CNS cells, infiltrating immune populations, and peripheral lymphoid tissues.

Taken together, current evidence suggests that epitope spreading is unlikely to be confined to a single anatomical location. Rather, distinct stages of autoreactive diversification may occur across multiple compartments. Within the CNS, tissue injury provides a source of newly available antigens and local antigen presentation by microglia, infiltrating macrophages, dendritic cells, and potentially other resident cells. CNS-draining lymph nodes provide an environment in which CNS-derived antigens can be presented to naïve or memory lymphocytes and where new autoreactive responses may emerge. Secondary lymphoid organs support germinal-centre reactions, affinity maturation, and the expansion of antigen-experienced B- and T-cell populations. Together, these compartments form a connected network in which antigen availability, antigen presentation, and lymphocyte activation occur in distinct but interacting anatomical niches (**Table 1**).

**Table 1:** Anatomical compartments potentially contributing to epitope spreading in MS.

Anatomical site	Antigen source	Major APCs	Relevant lymphocyte interactions	Potential relevance of EBI2
CNS lesions	Myelin debris, damaged oligodendrocytes, released CNS antigens	Microglia, infiltrating macrophages, dendritic cells	Local activation/reactivation of autoreactive T cells; recruitment of inflammatory cells	May regulate migration of encephalitogenic lymphocytes into CNS and influence localisation of infiltrating immune cells
CNS-draining lymph nodes	CNS-derived antigens transported from inflamed tissue	Dendritic cells, macrophages, B cells	Priming and expansion of autoreactive T- and B-cell responses; possible site of intermolecular spreading	EBI2 regulates positioning of APCs and lymphocytes within lymphoid tissue microenvironments
Secondary lymphoid organs	Peripheral antigens and CNS-derived antigens reaching lymphoid tissues	Dendritic cells, B cells	Germinal-centre reactions, affinity maturation, clonal expansion	Established role in B-cell, T-cell and dendritic-cell positioning; may influence frequency of antigen-specific cellular interactions
Tertiary lymphoid structures (if present)	Local CNS antigens	B cells, follicular dendritic cells, APCs	Local antibody responses and ongoing antigen presentation	Possible but currently poorly defined role

## 4 Drivers of epitope spreading in MS

Several factors determine whether newly available antigens are incorporated into the autoreactive repertoire. Beyond antigen availability itself, inflammatory cytokines, antigen-presenting cell activity, B-cell responses, and environmental influences shape the diversification of immune responses in MS and its experimental models. The efficiency of this process is influenced by the inflammatory milieu, particularly cytokines associated with Th1 and Th17 responses. These pathways have been strongly implicated in the expansion of autoreactive T-cell populations and are associated with relapses and disease activity (11). IL-23 signalling, in particular, plays a key role in maintaining pathogenic Th17 responses, and its blockade suppresses both disease progression and the emergence of new autoreactive specificities (21).

APCs are critical for initiating and sustaining epitope spreading. Dendritic cells, macrophages and microglia not only present newly available epitopes but also shape the quality of the immune response through co-stimulatory signals and cytokine production. Experimental manipulation of co-stimulatory pathways demonstrates this clearly. Blockade of CD28–B7 interactions prevents relapses and limits epitope spreading in EAE, while inhibition of CD40–CD154 signalling produces similar effects (22, 23). These findings indicate that T-cell activation thresholds are a key determinant of whether new autoreactive clones are recruited.

B cells also contribute to epitope spreading through antigen presentation and antibody production. In chronic EAE, both inter- and intramolecular spreading involve autoreactive B cells, suggesting that humoral responses can amplify antigen diversification (18). The presence of anti-myelin antibodies in relapsing models further supports a role for B cells in sustaining and expanding immune responses to CNS antigens. Beyond antibody production, B cells are particularly relevant to the spatial model of epitope spreading because they act as antigen-presenting cells and participate in germinal-centre reactions where antigen selection, clonal expansion, and affinity maturation occur. The anatomical positioning of B cells within these microenvironments influences which antigens are encountered and presented to T cells. Consequently, changes in B-cell localisation may affect not only the magnitude of immune responses but also the diversification of antigen specificity over time.

Evidence from B-cell repertoire studies further supports the concept that antigen-experienced B-cell populations undergo ongoing diversification in MS. Analyses of cerebrospinal fluid and peripheral blood have demonstrated clonal expansion, somatic hypermutation, and the presence of related B-cell clones across compartments, consistent with ongoing antigen-driven selection (24). Single-cell transcriptomic and B-cell receptor (BCR) repertoire analyses in relapsing–remitting MS identified distinct populations of somatically hypermutated memory B cells and antibody-secreting cells, indicating continued maturation of B-cell responses during disease. Notably, Ramesh et al. (24) reported increased expression of GPR183 (EBI2) within memory B-cell populations characterised by extensive somatic hypermutation, linking EBI2 expression to antigen-experienced B-cell states in a disease-relevant context. While these findings do not demonstrate a direct role for EBI2 in epitope spreading, they support the possibility that EBI2-expressing memory B cells participate in immune processes associated with autoreactive diversification.

Among environmental factors, Epstein–Barr virus (EBV) has received particular attention because strong epidemiological and mechanistic evidence links prior infection to MS development (25, 26). EBV persists within memory B cells, creating a potential connection between viral immunity, B-cell biology, and autoreactive responses (26–28). Molecular mimicry and cross-reactive immune responses involving EBV antigens may contribute to the initial activation of autoreactive lymphocytes (26, 29). However, within the framework proposed here, the relevance of EBV extends beyond molecular mimicry alone (30). Because EBV resides within B-cell populations that participate in antigen presentation, germinal-centre reactions, and affinity maturation, it may influence the cellular environments in which autoreactive diversification occurs. Whether EBV contributes directly to epitope spreading or instead modifies the conditions that permit diversification remains unresolved (31).

Finally, the balance between pro-inflammatory and regulatory signals influences the outcome of epitope spreading. While Th1 and Th17 responses promote the expansion of autoreactivity, regulatory pathways can limit or redirect this process. Interventions that increase IL-10 or shift immune responses towards a Th2 phenotype reduce inflammation and antigen presentation, thereby constraining the emergence of new epitopes (13, 32). These observations reinforce the idea that epitope spreading reflects the overall state of immune activation rather than a fixed pathogenic pathway (**Table 2**).

**Table 2:** Evidence supporting mechanisms potentially contributing to epitope spreading in MS.

Topic	Evidence from EAE	Evidence from human MS	Major limitation
Classical epitope spreading	Strong	Moderate/heterogeneous	Patient variability
B-cell-mediated diversification	Strong	Strong evidence for B-cell involvement; relationship to epitope spreading remains incompletely defined	Antigen specificity incompletely defined
EBV-associated mechanisms	Limited experimental evidence	Strong epidemiological and mechanistic evidence	Causality difficult to establish
CNS-compartmentalised immunity	Strong	Increasing evidence	Difficult to study longitudinally
EBI2-dependent immune positioning	Strong mechanistic evidence	Emerging evidence	No direct evidence linking EBI2 to epitope spreading

## 5 EBI2 as a regulator of immune cell positioning in epitope spreading

**Figure 1:**
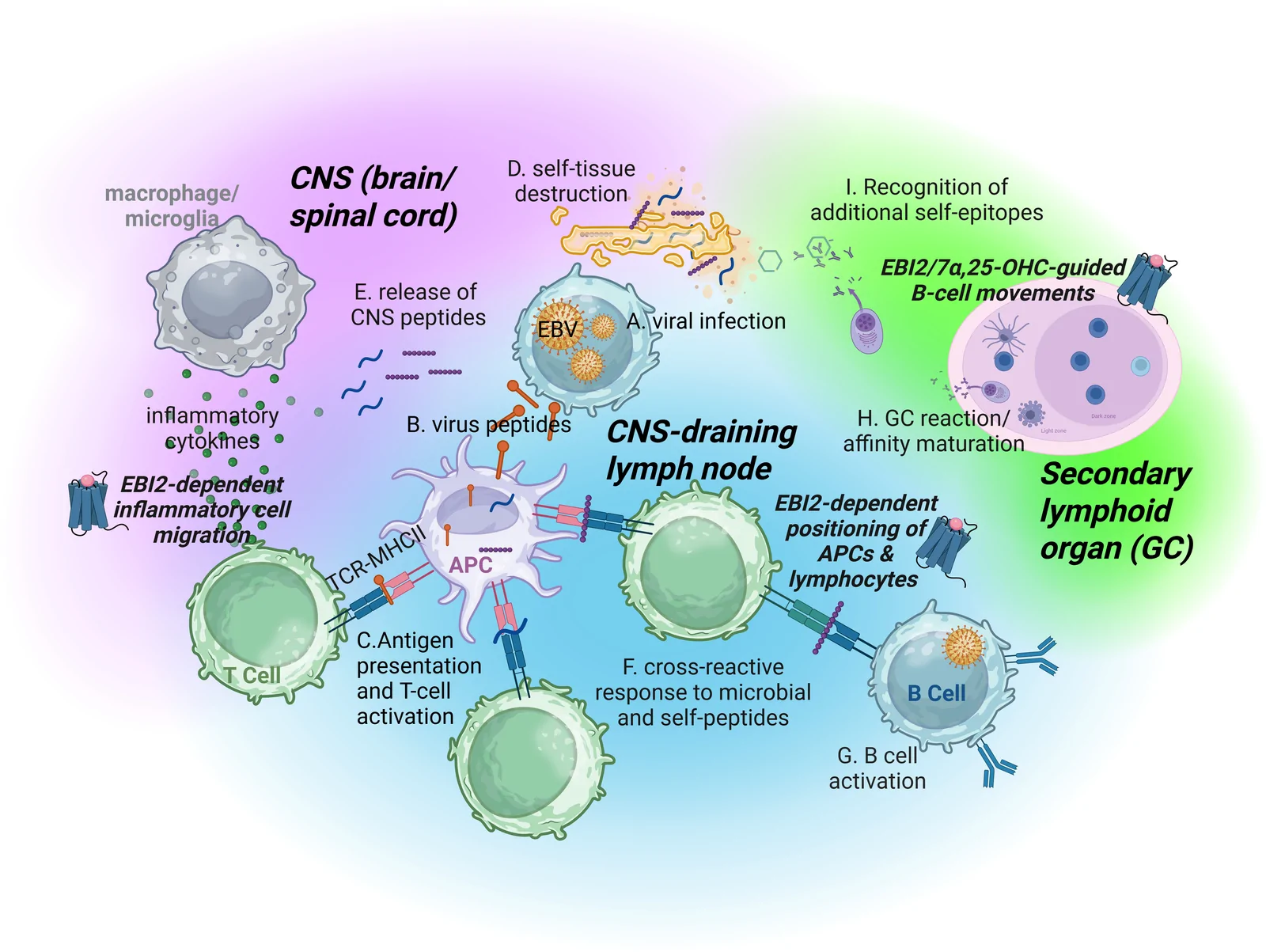
Spatial framework of epitope spreading in multiple sclerosis. The figure illustrates how autoreactive diversification may emerge through interactions occurring across three interconnected anatomical compartments: the CNS, CNS-draining lymph nodes, and secondary lymphoid organs. **(A)** EBV establishes persistent infection within B-cell populations, providing a source of viral antigens. **(B)** Viral antigens are processed into peptides that can be presented by APCs. **(C)** Antigen presentation promotes activation of antigen-specific T-cell responses. **(D)** Activated immune cells migrate in EBI2-dependent manner to the CNS and contribute to inflammatory tissue damage within the CNS. **(E)** Tissue injury results in the release of previously sequestered CNS-derived antigens, which become available for immune recognition. **(F)** Immune responses initially directed against microbial antigens may extend to structurally related self-antigens through cross-reactivity. Cross-reactive responses may provide an initial source of autoreactivity that subsequently broadens through epitope spreading. **(G)** Antigen-specific B cells are activated and participate in antigen presentation and antibody responses. **(H)** Germinal-centre reactions within lymphoid tissues support affinity maturation, clonal expansion, and selection of antigen-experienced B-cell populations. **(I)** Over time, immune responses diversify to include additional self-antigens, resulting in the expansion of autoreactive repertoires. EBI2-dependent immune-cell positioning is proposed to influence the spatial organisation of these processes. By regulating the localisation of B cells, antigen-presenting cells, and activated lymphocytes within lymphoid tissues, EBI2 may affect where antigen encounter, antigen presentation, and lymphocyte activation occur. EBI2-dependent migration may also contribute to the recruitment of immune cells into the inflamed CNS, where oxysterol gradients are increased during neuroinflammation. Under the model proposed in this Review, EBI2 is not considered a direct driver of epitope spreading but rather a candidate regulator of the anatomical conditions that may permit the emergence of new autoreactive specificities.

The generation of new autoreactive responses during epitope spreading depends not only on antigen availability and immune activation but also on the spatial organisation of immune cells. EBI2 (GPR183), an oxysterol-sensing G protein-coupled receptor, plays a central role in regulating the migration and positioning of immune cells within lymphoid tissues and inflamed sites, placing it in a position to influence processes underlying epitope spreading (**Figure 1**). While EBI2 has established roles in immune-cell migration and positioning, no study has directly demonstrated that EBI2 regulates epitope spreading in MS or EAE. The discussion below therefore distinguishes established functions of EBI2 from their potential implications for autoreactive diversification.

Although a direct genetic association between EBI2 and MS susceptibility has not been established, several studies implicate this receptor in disease-relevant pathways. In MS lesions, increased EBI2 expression has been reported primarily in infiltrating immune cells and resident glial populations, including inflammatory T cells and myeloid cells, rather than being specifically demonstrated in lesion-associated B cells (33, 34). In experimental models, EBI2 expression is upregulated during the induction phase of EAE and promotes the migration of activated lymphocytes into the CNS along gradients of its ligand, 7α,25-dihydroxycholesterol (7α,25-OHC) (33). Consistent with this, CH25H-deficient mice, which have reduced levels of this oxysterol, show attenuated disease associated with decreased infiltration of CD4+ T cells into the CNS (35). Thus, current lesion-based evidence supports a role for EBI2 in inflammatory cell migration and CNS immune-cell positioning, but does not establish that EBV-infected B cells express elevated EBI2 within MS lesions.

In addition to its role in immune cell trafficking, EBI2 is expressed in oligodendrocytes and has been linked to myelination and remyelination. Experimental studies have shown that EBI2 contributes to myelin development and can limit demyelination in ex vivo and *in vivo* models (36–38). These findings suggest that EBI2 may influence both inflammatory and repair processes within the CNS.

A key function of EBI2 is the regulation of immune cell positioning within secondary lymphoid organs. The receptor is highly expressed in B cells, T cells and dendritic cells and is rapidly upregulated upon activation (39). Consistent with this, single-cell transcriptomic analyses of patients with relapsing–remitting MS have identified GPR183 expression in somatically hypermutated memory B-cell populations, further supporting a potential association between EBI2 signalling and antigen-experienced B-cell states (24). By sensing gradients of 7α,25-OHC, EBI2 directs the migration of these cells to specific microenvironments, including the interfollicular regions and marginal zone bridging channels, where antigen presentation and lymphocyte activation occur (40, 41). Disruption of EBI2 signalling impairs the localisation of dendritic cells and reduces their ability to capture and present antigen, leading to diminished T-cell activation and B-cell responses (42, 43). The proposed connection between EBV, B cells and EBI2 is therefore most coherent in lymphoid or antigen-experienced B-cell compartments, where EBI2 is known to regulate B-cell positioning, rather than as a demonstrated feature of EBV-infected B cells within MS lesions.

These spatial effects are particularly relevant in the context of epitope spreading. Efficient spreading requires repeated encounters between antigen-presenting cells and lymphocytes, as well as access to newly available antigens. Because EBI2 regulates the positioning of B cells, dendritic cells, and activated lymphocytes within lymphoid tissues, it may influence the frequency and location of these interactions. However, direct evidence linking EBI2 signalling to epitope spreading is currently lacking. I therefore propose that EBI2 could regulate the conditions that permit the emergence of new autoreactive specificities rather than acting as a demonstrated driver of epitope spreading itself. Under this model, altered positioning of antigen-presenting cells and lymphocytes may affect germinal centre dynamics, antigen presentation, and the recruitment of additional autoreactive clones, but these possibilities remain to be tested experimentally. As summarised in **Table 1**, EBI2 could theoretically influence immune-cell interactions across multiple anatomical compartments relevant to epitope spreading.

Beyond classical lymphocyte populations, EBI2 is also expressed in innate lymphoid cells (ILC3s), which have been shown to participate in neuroinflammation. A circulating population of inflammatory ILC3s can infiltrate the CNS and present antigen to myelin-reactive T cells during EAE, contributing to disease progression (44). Given the established role of EBI2 in ILC3 migration in peripheral tissues, it is plausible that this pathway also contributes to the positioning of these cells during CNS inflammation.

Finally, interactions between peripheral and CNS immune compartments may further influence epitope spreading. T cells primed in gut-associated lymphoid tissue acquire homing markers such as α4β7 integrin, and changes in the distribution of these cells have been observed under inflammatory conditions (45, 46). These findings raise the possibility that immune imprinting outside the CNS may shape the repertoire of cells available for recruitment during MS and thereby influence the pattern of epitope spreading.

Taken together, EBI2 emerges as a regulator of the spatial context in which immune responses unfold. Rather than directly initiating autoreactivity, it influences the efficiency and location of antigen presentation and lymphocyte activation. In MS, this may determine whether newly released epitopes are ignored or incorporated into an expanding autoreactive response.

## 6 Therapeutic implications and limitations of targeting epitope spreading

The concept of epitope spreading has important implications for the development of therapeutic strategies in multiple sclerosis. If disease progression involves the gradual expansion of autoreactivity to multiple antigens, then approaches targeting a single dominant epitope are unlikely to provide durable benefit. Instead, effective therapies may need to account for the dynamic and evolving nature of antigen recognition.

Experimental studies support this view. Interventions that broadly suppress T-cell activation or co-stimulatory signalling can limit both disease progression and the emergence of new autoreactive specificities. Blockade of CD28–B7 interactions or CD40–CD154 signalling prevents relapses and reduces epitope spreading in EAE models (22, 23). Similarly, modulation of cytokine pathways that sustain pathogenic T-cell responses, such as IL-23 signalling, attenuates disease and constrains the diversification of autoreactivity (21).

More targeted approaches have focused on inducing antigen-specific tolerance. Tolerogenic dendritic cells loaded with myelin antigens reduce disease severity and limit spreading in EAE, suggesting that restoring immune tolerance remains a viable strategy (47). Antigen-coupled cells and nanoparticle-based delivery systems have also been shown to induce antigen-specific tolerance and suppress disease progression (48). However, these approaches face a fundamental limitation: the identity and hierarchy of relevant epitopes vary between patients and change over time, making it difficult to define appropriate targets.

This limitation has led to the development of multi-epitope strategies. Administration of peptide cocktails derived from myelin proteins can prevent disease induction and reduce autoreactive T-cell responses in EAE (49). More refined approaches using engineered multi-epitope constructs show improved efficacy and longer-lasting immunomodulatory effects, likely because they account for the diversity of antigenic targets involved in disease (50). These findings suggest that therapeutic success may depend on addressing antigenic diversity rather than focusing on individual epitopes.

Additional experimental approaches further support this concept. Homotaurine treatment limits Th17- and Th1-driven spreading within the CNS and reduces disease severity (51). Inhibition of PARP-1 similarly suppresses epitope spreading and promotes the induction of regulatory immune responses (52). These interventions do not target specific antigens but instead modify the conditions that permit the expansion of autoreactivity.

Clinical evidence, although limited, is consistent with these observations. Autologous hematopoietic stem cell transplantation has been associated with long-term suppression of disease activity and has been reported to reduce epitope spreading in a small cohort of patients (53). While such approaches are not widely applicable, they provide proof of principle that resetting the immune system can constrain the diversification of autoreactive responses.

Despite these advances, several challenges remain. Epitope spreading is difficult to measure directly in patients, and its contribution to disease progression likely varies between individuals. In addition, spreading is not strictly required for disease, as demonstrated in experimental models where progression occurs in its absence (19). This suggests that targeting epitope spreading alone may not be sufficient and that therapies must also address parallel pathogenic mechanisms.

The spatial framework proposed here also raises the possibility that therapeutic interventions could target the cellular interactions that permit autoreactive diversification rather than individual antigenic targets. In principle, modulation of immune-cell positioning could reduce the likelihood that newly available CNS antigens are incorporated into the autoreactive repertoire. Because EBI2 regulates the localisation of B cells, dendritic cells, and activated lymphocytes, this pathway represents a candidate mechanism through which such effects might be achieved.

However, the therapeutic implications of EBI2 modulation are not straightforward and represent a central unresolved question arising from the present framework. Inhibition of EBI2 signalling may limit inflammatory immune-cell migration and reduce opportunities for autoreactive diversification, whereas activation of EBI2 has been associated with remyelination and tissue repair in experimental models. Taken together, these observations highlight a potential therapeutic paradox. One possibility is that the outcome of EBI2 activation may depend not only on receptor signalling itself but also on the spatial distribution of its ligand. In principle, exogenous administration of 7α,25-OHC could alter endogenous oxysterol gradients and thereby impair directional migration of EBI2-expressing immune cells, even while maintaining receptor activation. Under such conditions, EBI2 activation might support remyelination and repair while simultaneously reducing the efficiency of immune-cell recruitment into inflamed tissues. Although this possibility remains speculative and has not been directly tested in MS, it illustrates how the spatial organisation of EBI2 signalling may be as important as the magnitude of receptor activation. The net effect of EBI2 modulation is therefore likely to depend on disease stage, cellular target, anatomical compartment, and the local distribution of oxysterol ligands. Future therapeutic strategies may therefore require cell-type-specific or temporally defined approaches rather than uniform activation or inhibition of the pathway.

Taken together, these findings indicate that epitope spreading should be viewed as a feature of an active and evolving immune response rather than a single therapeutic target. Strategies that limit immune activation, restrict antigen presentation, or restore tolerance across multiple epitopes are more likely to achieve sustained disease control than those focused on individual antigens.

## 7 Testable predictions arising from the spatial EBI2 model

The model proposed here is inferential and requires direct experimental validation. No study has yet shown that genetic or pharmacological manipulation of EBI2 alters epitope spreading in MS or EAE. However, the spatial framework generates several testable predictions. First, EBI2 deficiency or antagonism would be expected to alter the anatomical distribution of antigen-presenting cells and activated lymphocytes in CNS-draining lymph nodes and inflamed CNS tissue, potentially reducing the recruitment of new autoreactive T- or B-cell specificities. Second, longitudinal T-cell receptor (TCR) and BCR repertoire analyses during EAE could determine whether EBI2 signalling influences the emergence of secondary myelin-reactive clones. Third, if EBI2-expressing memory B cells contribute to antigenic diversification in MS, these cells should show evidence of clonal expansion, somatic hypermutation, and antigen-experienced transcriptional programmes in patient CSF or CNS-associated compartments. Finally, therapeutic manipulation of EBI2 should be evaluated in a stage- and cell-type-specific manner, because inhibition of EBI2 may limit inflammatory immune-cell positioning whereas activation may support remyelination and repair. Future studies will need to determine whether these functions can be separated therapeutically or whether they represent an unavoidable trade-off of targeting this pathway. Thus, the key question is not simply whether EBI2 should be blocked or stimulated, but when, where, and in which cell population this pathway is being targeted.

## 8 Conclusion

Epitope spreading provides a framework for understanding how immune responses in multiple sclerosis evolve over time. Evidence from experimental models and clinical observations indicates that autoreactivity is not fixed but expands to include new antigenic targets as disease progresses. This process is shaped by tissue damage, inflammatory signalling, antigen presentation, and the spatial organisation of immune cells. At the same time, evidence for epitope spreading in human MS remains heterogeneous, and disease progression can occur in its apparent absence in some experimental settings. Epitope spreading should therefore be viewed as one potential contributor to disease evolution rather than a universal explanation for chronic neuroinflammation.

Within this context, EBI2 emerges as a candidate regulator of immune-cell positioning that may influence the likelihood of epitope spreading by controlling where and how immune interactions occur. However, the proposed relationship between EBI2 and autoreactive diversification remains hypothetical and requires direct experimental validation.

The implications of this evolving autoreactivity extend beyond questions of disease mechanism. Therapeutic strategies that fail to consider the evolving nature of antigen recognition may therefore be inherently limited. A better understanding of how autoreactive repertoires change over time, where this diversification occurs in human MS, and how it is regulated will be critical for the development of more effective and durable treatments. In particular, it remains unclear whether immune-cell positioning actively determines the emergence of new autoreactive specificities, and whether pathways such as EBI2 signalling can be manipulated to limit autoreactive diversification without compromising tissue repair. Addressing these questions will be essential for determining whether the spatial organisation of immune responses represents a tractable target for future intervention.
