# Correction: Epitope spreading in multiple sclerosis is shaped by the spatial organisation of immune responses

**DOI:** 10.3389/fimmu.2026.1953873

**Published:** 2026-08-14

**Authors:** Aleksandra Rutkowska

**Affiliations:** Department of Anatomy and Neurobiology, Rutkowska Lab, Medical University of Gdańsk, Gdansk, Poland

**Keywords:** 7α,25-Dihydroxycholesterol (7α,25-OHC), B cells, EBI2 (GPR183), epitope spreading, Epstein–Barr virus, germinal centres, immune cell migration, multiple sclerosis

The reference for “38” was erroneously written as “Konieczna-Wolska K, Caratis F, Opiełka M, Biernacki K, Urbanowicz K, Klimaszewska J, et al. Accelerated remyelination and immune modulation by the EBI2 agonist 7a,25-dihydroxycholesterol analogue in the cuprizone model. BioMed Pharmacother. (2024) 181(3):5173–88. doi: 10.1016/ J.BIOPHA.2024.117653”. It should be “Konieczna-Wolska K, Caratis F, Opiełka M, Biernacki K, Urbanowicz K, Klimaszewska J, Pobiarzyn P, Krajewski O, Demkowicz S, Smoleński RT, Karaszewski B, Seuwen K, Rutkowska A. Accelerated remyelination and immune modulation by the EBI2 agonist 7α,25-dihydroxycholesterol analogue in the cuprizone model. Biomed Pharmacother. 2024 Dec;181:117653. doi: 10.1016/j.biopha.2024.117653”.

The original version of this article has been updated.

The reference for “6” was erroneously written as “Kanduc D, Shoenfeld Y. From anti-EBV immune responses to the EBV diseasome via cross-reactivity. (2020) 7(2):51–63. doi: 10.1055/S-0040-1715641”. It should be “Kanduc D, Shoenfeld Y. From Anti-EBV Immune Responses to the EBV Diseasome via Cross-reactivity. Glob Med Genet. 2020 Aug;7(2):51-63. doi: 10.1055/s-0040-1715641”.

The original version of this article has been updated.

